# Clinical Relevance of Galectin-1 and Galectin-3 in Rheumatoid Arthritis Patients: Differential Regulation and Correlation With Disease Activity

**DOI:** 10.3389/fimmu.2018.03057

**Published:** 2019-01-09

**Authors:** Santiago P. Mendez-Huergo, Pablo F. Hockl, Juan C. Stupirski, Sebastián M. Maller, Luciano G. Morosi, Nicolás A. Pinto, Ana M. Berón, Jorge L. Musuruana, Gustavo G. Nasswetter, Javier A. Cavallasca, Gabriel A. Rabinovich

**Affiliations:** ^1^Laboratorio de Inmunopatología, Instituto de Biología y Medicina Experimental (IBYME), Consejo Nacional de Investigaciones Científicas y Técnicas (CONICET), Buenos Aires, Argentina; ^2^División Reumatología, Hospital de Clínicas “José de San Martín”, Universidad de Buenos Aires, Buenos Aires, Argentina; ^3^Sección de Reumatología y Enfermedades Autoinmunes Sistémicas, Hospital “José Bernardo Iturraspe”, Santa Fe, Argentina; ^4^Departamento de Química Biológica, Facultad de Ciencias Exactas y Naturales, Universidad de Buenos Aires, Buenos Aires, Argentina

**Keywords:** rheumatoid arthritis, galectin-1, galectin-3, inflammation, autoimmune disease

## Abstract

Galectins, a family of animal lectins, play central roles in immune system regulation, shaping both innate and adaptive responses in physiological and pathological processes. These include rheumatoid arthritis (RA), a chronic multifactorial autoimmune disease characterized by inflammatory responses that affects both articular and extra-articular tissues. Galectins have been reported to play central roles in RA and its experimental animal models. In this perspective article we present new data highlighting the regulated expression of galectin-1 (Gal-1) and galectin-3 (Gal-3) in sera from RA patients under disease-modifying anti-rheumatic drugs (DMARDs) and/or corticoid treatment in the context of a more comprehensive discussion that summarizes the roles of galectins in joint inflammation. We found that Gal-1 levels markedly increase in sera from RA patients and positively correlate with erythrocyte sedimentation rate (ERS) and disease activity score 28 (DAS-28) parameters. On the other hand, Gal-3 is downregulated in RA patients, but positively correlates with health assessment questionnaire parameter (HAQ). Finally, by generating receiver-operator characteristic (ROC) curves, we found that Gal-1 and Gal-3 serum levels constitute good parameters to discriminate patients with RA from healthy individuals. Our findings uncover a differential regulation of Gal-1 and Gal-3 which might contribute to the anti-inflammatory effects elicited by DMARDs and corticoid treatment in RA patients.

## Introduction

Rheumatoid arthritis (RA) is a highly prevalent chronic disease with multifactorial etiology. It is characterized by generalized inflammation in multiple joints, leading to cartilage and bone erosion and articular deformation. The disease comprises a complex interaction between genetic susceptibility and environmental stimuli, including epigenetic modifications (1). Galectins have emerged as master regulators of immune system homeostasis, playing key roles in the amplification and/or resolution of inflammatory processes, including RA (2, 3).

## Galectins in Inflammation

Galectins are soluble lectins defined by their affinity toward galactose-β1-4-*N*-acetylglucosamine (*N*-acetyl-lactosamine, LacNAc)-enriched glycoconjugates present on the cell surface or extracellular matrix. Until now, 15 galectins have been described in vertebrates and classified into three groups according to their molecular architecture: (1) “proto-type” galectins (e.g., Gal-1), consisting of only one carbohydrate recognition domain (CRD) which can homodimerize; (2) “tandem-repeat” galectins (e.g., Gal-8 and−9), which present two different CRDs in tandem connected by a short peptide; and (3) the “chimera-type” galectin, Gal-3, consisting of one CRD connected to a non-lectin N-terminal region that supports oligomerization (4, 5). The glycan-binding specificities of individual members of the galectin family have been extensively discussed recently (4).

Although some galectins exhibit a broad tissue localization (e.g., Gal-1 and Gal-3), others show a selective distribution pattern (2). Whereas some members of the galectin family trigger anti-inflammatory responses and serve as pro-resolving mediators, others display pro-inflammatory activity enhancing innate and adaptive immunity (6). Thus, the functional outcome of galectin signaling may differ greatly, depending on the particular galectin involved, the number and branching of specific glycans serving as potential ligands and the biochemical nature of these multivalent interactions (4, 7). In this regard, inflammation induces changes in the glycosylation signature of both immune cells and inflamed tissue, leading to either masking or unmasking of galectin-specific glycoepitopes (4, 8). Particularly, LacNAc residues recognized by Gal-1, which are present on the branches of *N*- or *O-*linked glycans, are created by the concerted action of specific glycosyltransferases including the N-acetylglucosaminyl transferase 5 (MGAT5), an enzyme that generates β1-6-N-acetylglucosamine branches in complex *N-*glycans, and the core 2 β1-6-N-acetylglucosaminyl transferase 1 (C2GNT1), which acts on asialo-galactose-β1-3-N-acetylgalactosamine core 1 *O*-glycans to synthetize the core 2 branching structure (4). Since Gal-1 and Gal-3 are ubiquitously expressed and display context-dependent functional roles, their immunomodulatory effects have been described in several inflammatory microenvironments (2).

Given the prominent expression of Gal-1 in tumors and immune privileged sites and its up-regulation during the recovery phase of autoimmune inflammation (9–13), this lectin has been proposed to play key roles in suppression of antitumor responses, maintenance of immune tolerance and resolution of chronic inflammation, acting as a novel regulatory checkpoint that links innate and adaptive responses (14). Gal-1 shapes immune responses by selectively deleting Th1 and Th17 effector cells (15), promoting a tolerogenic and pro-migratory dendritic cell (DC) phenotype (13, 16), fostering expansion of regulatory T cells (Tregs) (10, 17–19) and fine-tuning the function of neutrophils, monocytes and macrophages (20, 21). These broad immunoregulatory effects have been validated in several experimental models of autoimmunity, allergy, infection, and cancer (2, 7, 22–24).

On the other hand, Gal-3 has controversial pro- or anti-inflammatory activities depending on various factors including its intracellular or extracellular localization and the target cell implicated in these processes (25). Although it may contribute to resolution of inflammation by clearing apoptotic neutrophils (26), this lectin displays mostly pro-inflammatory effects by reinforcing activation of macrophages, DCs, mast cells, and natural killer cells, as well as T and B lymphocytes (27).

## Galectins in Rheumatoid Arthritis

Heritability of RA is calculated to be around 65%, with more than 100 RA-risk-associated genomic loci (28). A few polymorphisms in individual galectins that could be associated to progression or severity of RA have been described. *LGALS3* +*292C*, a polymorphism in the gene encoding Gal-3, is more common in RA patients (29). Moreover, a polymorphism in the gene encoding Gal-8 (rs2737713), generated by a missense mutation that changes a phenylalanine for tyrosine (F19Y), exhibits a strong association with RA in a Caucasian population (30). This mutation seemed to have no major effect on carbohydrate binding at least in solid-phase assays. Furthermore, a C3279T polymorphism in *LGALS2 gene* (encoding Gal-2), has been associated with diastolic blood pressure in RA patients at increased risk for hypertension (31).

**Figure 1:**
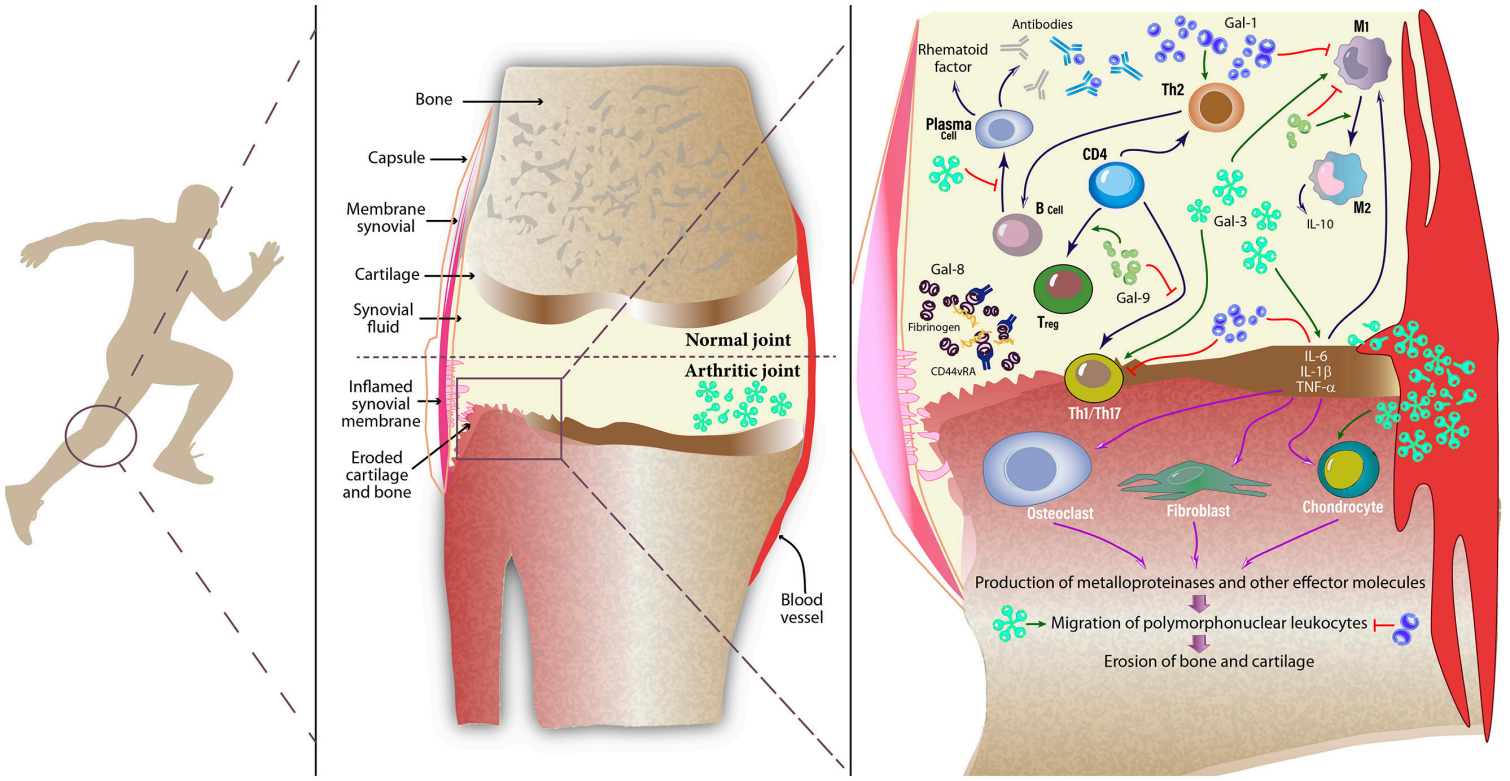
Role of galectins in inflamed synovial tissue. Galectins are expressed by a number of inflammatory cells (both innate and adaptive immune cells), endothelial cells, stromal cells, and synovial fibroblasts. These glycan-binding proteins influence a variety of cellular programs that control amplification and resolution of inflammatory responses. Galectins can behave as pro- or anti-inflammatory mediators by modulating the physiology of immune cells, including monocytes, macrophages, synovial fibroblasts, Th1, Th2, and Th17 cells, regulatory T (Treg) cells, B cells, neutrophils and mast cells. By positively or negatively regulating inflammation, galectins may directly or indirectly influence the clinical course of RA. While Gal-1 enhances a Th2-Treg response profile, polarizes macrophages toward an M2 phenotype and induces apoptosis of Th1 and Th17 cells, Gal-3 activates fibroblasts and induces secretion of pro-inflammatory cytokines. Circulating autoantibodies reduce effective Gal-1 concentrations in synovial fluid of patients with RA. On the other hand, Gal-9 controls CD4^+^ T cell functions through binding to TIM-3^+^ cells. Moreover, Gal-8 has pro-apoptotic and anti-inflammatory activity in the inflamed joint; however a soluble form of CD44 reduces availability of this tandem-repeat galectin by forming complexes with fibrinogen. Gal, Galectin; TNF, Tumor necrosis factor; IL, Interleukin; Th, T helper cell; Treg, regulatory T cells; M1, pro-inflammatory macrophage; M2, anti-inflammatory macrophage.

A common feature of RA is the altered hyper-activated state of the stromal tissue and the immune system (1). Changes in both innate and adaptive immune pathways are common findings in RA patients (32). Gal-3 has been identified as a pro-inflammatory mediator both in RA patients and animal models of the disease. Gal-3 mRNA and protein were detected at the synovial membrane, while Gal-3-binding protein was found to be predominantly expressed at sites of bone destruction (33). Interestingly, expression of Gal-1 was not found at sites of synovial fibroblast invasion in RA (33). Synovial fibroblasts from RA patients expressed higher levels of CD51 and CD61 integrins, which individually, or by forming the α_V_β_3_ complex (vitronectin receptor), binds to cartilage oligomeric matrix protein and induces secretion of Gal-3 (34). Externalization of this lectin influences the shape and persistence of joint inflammation by inducing local fibroblasts to secrete pro-inflammatory cytokines including IL-6, GM-CSF and MMP-3 and chemokines such as CCL2, CXCL8, CCL3, and CCL5 (35). Stimulation of IL-6 secretion by Gal-3 is mediated by Toll-like receptor-2,−3, and−4 in human synovial fibroblasts (36), contributing to amplification of pro-inflammatory responses (Figure 1).

Before the clinical onset of the disease, a “pre-RA” condition arises, which displays both immunologic and metabolic alterations (37). Follow-up studies in undifferentiated arthritis (UA) patients, naïve for both disease-modifying anti-rheumatic drugs (DMARDs) and corticosteroids, showed that serum Gal-3 levels are high in those patients that progress to RA after 1 year. Although serum Gal-3 was a poor prognostic marker itself, the combination with anti-cyclic citrullinated peptide (CCP) levels or bone marrow edema score could help categorize UA subsets at early phases (38). Moreover, in another study, serum Gal-3 levels showed no differences compared to controls in DMARDs- and corticosteroid-naïve patients with < 6 months of RA diagnosis, but were significantly elevated in anti-CCP positive vs. anti-CCP negative patients and healthy subjects (39). Furthermore, in a cohort of 20 RA patients serum Gal-3 levels positively correlated with those found in synovial fluid (33), suggesting possible association between systemic and local galectins.

Notably, autoantibodies that could reduce or block biological activities of galectins have been found in different settings. Xibillé-Friedmann and colleagues reported reduced Gal-1 levels in synovial fluid of RA patients due to the presence of anti-Gal-1 autoantibodies (40), a similar effect as that found in uveitis patients (41) (Figure 1). Moreover, autoantibodies against Gal-8 and Gal-9 have also been detected in RA patients (42, 43).

In a model of antigen-induced arthritis, Forsman and colleagues found that joint inflammation and bone erosion were attenuated, antigen-specific IgG and pro-inflammatory cytokines TNF-α and IL-6 were decreased, and the number of Th17 cells was significantly reduced in *Lgals3*^−/−^ vs. WT mice, suggesting a pathogenic role for this lectin in the development and progression of RA (44). In contrast, *Lgals1*^−/−^ mice developed a more severe inflammatory response in a model of collagen-induced arthritis (CIA) with higher penetrance and an accelerated clinical onset (45). In this regard, in early studies, we demonstrated the therapeutic potential of Gal-1 in the CIA model. Injection of syngeneic fibroblasts genetically engineered to secrete Gal-1, or daily administration of recombinant Gal-1 suppressed clinical and histopathological manifestations of arthritis and promoted a shift toward a Th2-mediated anti-inflammatory response (46). These findings were integrated by Wang et al. who successfully treated rats using lentiviral vectors aimed at overexpressing Gal-1 or silencing Gal-3, revealing broad anti-inflammatory responses characterized by improved radiographic and histological scores (47). Additionally, downregulation of Gal-1 and upregulation of Gal-3 expression were found in synovial tissue from patients with juvenile idiopathic arthritis (48, 49).

Interestingly, Eshkar-Sebban et al. found that a CD44 variant expressed in synovial fluid of RA patients -CD44vRA- sequesters Gal-8 by forming a soluble complex with fibrinogen, thus reducing the availability of this lectin in the inflamed joint (50). Furthermore, elevated levels of Gal-9 were detected in synovial fluid from patients, an effect that was accompanied by a higher percentage of Gal-9-positive cells in synovial tissue (51). By using a stable mutant protein resistant to proteolysis, Seki et al. showed that Gal-9, but not Gal-1,−3, or−8, induced apoptosis of fibroblast-like synoviocytes (51). Later, the authors found that Gal-9 suppressed clinical manifestations of CIA by reducing the synthesis of pro-inflammatory cytokines IL-17, IL-12, and IFN-γ in the joints and lowering the number of CD4^+^ T cells expressing T-cell immunoglobulin and mucin-domain containing-3 (TIM-3) in peripheral blood (52). Nonetheless, this effect was impaired in RA patients due to reduced TIM-3 expression (53). Furthermore, Gal-9 also reduced the severity of immune complexes-induced arthritis by downregulating FcγRIII and upregulating FcγRIIb in macrophages, an effect that ultimately led to IL-10 secretion and inhibition of TNF-α and IL-1β production (54). Mechanistically, Gal-9 acted by inducing the *in vitro* differentiation of Tregs, while suppressed polarization toward a Th17 phenotype (52). In contrast, a recent study suggested a rather pro-inflammatory role of Gal-9, as intra-articular injection of this lectin facilitated mononuclear cell migration and favored arthritogenic responses (55). Thus, the coordinated action and differential regulation of individual members of the galectin family will finally dictate clinical responses in RA patients (Figure 1).

## Clinical Relevance of Gal-1 And Gal-3 in Patients With Rheumatoid Arthritis

Based on its broad anti-inflammatory activity, we evaluated Gal-1 serum levels in patients with established RA (defined by the American College of Rheumatology 2010 classification criteria). We recruited 32 patients and 19 sex- and age-matched healthy volunteers from Hospital de Clínicas “José de San Martín” (Buenos Aires, Argentina) (Table 1). Patients ranged from 1 to 28 years since RA was first diagnosed and were all under treatment with at least one DMARD, mainly methotrexate. Determination of Gal-1 was performed using an in-house ELISA as described (56). Detailed description of Materials and methods is shown as Supplementary Data.

**Table 1:** Demographic, clinical, and laboratory characteristics of patients with RA.

	**Cohort 1 (n:32)**	**Cohort 2 (n:48)**
**Gender**		
Female	29	46
Male	3	2
**Age, median years (range)**	41 (24–64)	48 (30–67)
**RA duration, mean years (range)**	7.8 (1–28)	9.1 (1–28)
**Disease activity parameters**		
Functional Class		
Class I	4/32 (12.6%)	20/48 (41.7%)
Class II	14/32 (43.7%)	21/48 (43.7%)
Class III	11/32 (34.3%)	5/48 (10.4%)
N/A	3/32 (9.4%)	2 (4.2%)
DAS-28, mean (range)	4.4 (1.75–8)	4.4 (1.96–6.28)
HAQ-A, mean (range)	1.30 (0.25–2.25)	1.27 (0–4.12)
VAS, mean (range)	41.4 mm (0–100)	37.1 mm (0–100)
ESR, mean (range)	27.7 mm (10–91)	32.6 mm (5–68)
**Serology**		
RF			
Positive	28	38
Negative	0	7
N/A	4	3
Anti-CCP		
Positive	18	14
Negative	1	0
N/A	12	34
**Treatment**			
Methotrexate	26/32 (81.3%)	40/48 (83.3%)
Corticosteroids	19/32 (59.4%)	43/48 (89.6%)
HCQ/CQ	9/32 (28.1%)	19/48 (39.6%)
Sulfasalazine	1/32 (3.1%)	4/48 (8.3%)
Leflunomide	1/32 (3.1%)	11/48 (23%)
Anti-TNFα	6/32 (18.8%)	2/48 (4.2%)
Other biologicals (rituximab, abatacept)	2/32 (6.3%)	0/48 (0%)
NSAIDs	9/32 (28.1%)	33/48 (68.8%)
Other	9/32 (28.1%)	4/48 (8.3%)
N/A	3/32 (9.4%)	3/48 (6.2%)

*N/A, Not Available; RF, Rheumatoid Factor; anti-CCP, anti-cyclic citrullinated peptide; NSAIDs, non-steroidal anti-inflammatory drugs. Others: folic acid, VitD3, risedronate, calcium*.

**Figure 2:**
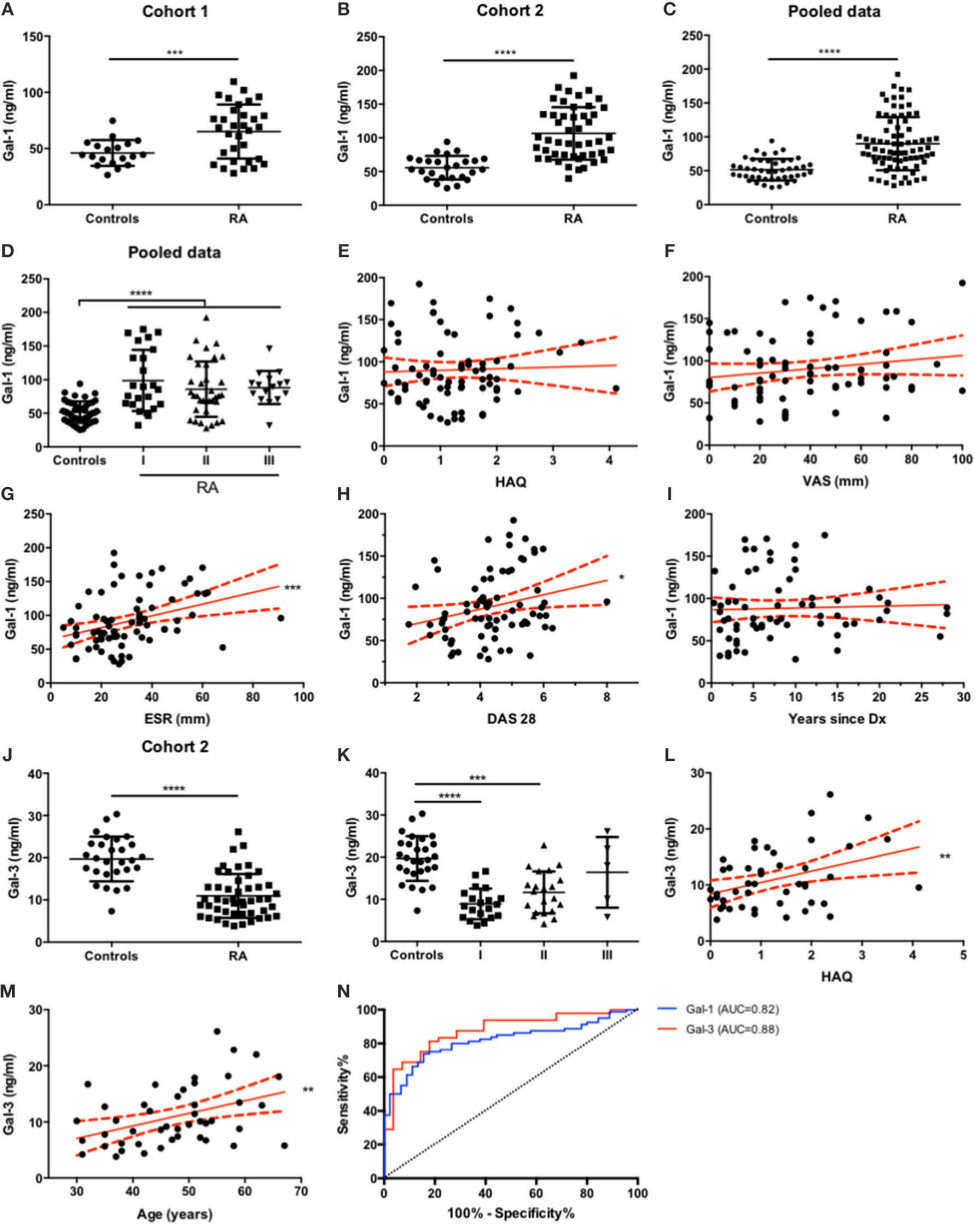
Serum Gal-1 and Gal-3 expression levels discriminate RA patients from healthy individuals. **(A-C)**. Determination of serum Gal-1 levels (ELISA) in controls and RA patients from cohort 1 **(A)**, cohort 2 **(B)** and pooled data **(C)**. **(D)**. Gal-1 serum levels from all patients **(C)** classified by functional status. **(E-I)**. Correlation analysis of Gal-1 serum levels of all patients with HAQ **(E)**, VAS **(F)**, ESR **(G)**, DAS-28 **(H)** and RA duration **(I)**. **(J)**. Determination of serum Gal-3 levels (ELISA) in controls and RA patients from cohort 2. **(K)**. Gal-3 serum levels of RA patients from cohort 2 **(J)** classified by functional status. **(L-M)**. Correlation analysis of Gal-3 serum levels of RA patients from cohort 2 with HAQ **(L)** and age **(M)**. **(N)**. ROC curve analysis to assess Gal-1 (blue) and Gal-3 (red) capacity to discriminate between RA patients and healthy individuals. *p < 0.05, **p < 0.01, ***p < 0.001. ****p < 0.0001. All variables analyzed were tested for Gaussian distribution with D'Agostino and Pearson omnibus normality test. For comparisons between two groups, unpaired *t* test with Welch's correction or Mann-Whitney tests were applied as appropriate. For comparisons between more than two groups, Kruskal-Wallis test was applied. For correlation analysis, Pearson or Spearman correlation tests were applied as appropriate. To determine the capability of Gal-1 and Gal-3 serum level measurements to discriminate between RA patients and controls, ROC curves were generated.

Analysis of circulating Gal-1 showed significantly higher levels of this lectin in serum obtained from RA patients compared to control individuals (Figure 2A). To further validate these findings and given the lack of differences reported in another study (40), we recruited a second, independent and larger cohort of patients from Hospital “José Bernardo Iturraspe” (Santa Fe, Argentina). Twenty nine healthy volunteers and 48 RA patients under DMARD treatment were enrolled in the study. Cohort 2 validated our previous observation, as RA patients again showed significantly higher levels of serum Gal-1 compared to controls (Figure 2B).

Next, we explored the potential associations of Gal-1 with clinical parameters of disease activity. For this purpose, and to gain statistical robustness, we pooled data from both cohorts. Regardless of differences in the median Gal-1 serum levels between RA patients from cohort 1 (median = 68.77 ng/ml) and cohort 2 (median = 95.63 ng/ml), analysis of pooled data from both cohorts revealed, as expected, elevated Gal-1 levels in sera from RA patients compared to controls (Figure 2C). Based on this finding, we regrouped RA patients based on their functional status classification, and found that, compared to controls, serum Gal-1 levels were significantly increased in all functional classes; yet revealing no statistical differences (Figure 2D).

Next, we analyzed whether Gal-1 serum levels may correlate with quantitative parameters of disease activity derived from patients' questionnaires, such as VAS (Visual Analog Scale) and physical function such as HAQ (Health Assessment Questionnaire). As shown in Figures 2E,**F**, neither VAS nor HAQ parameters showed correlation with circulating Gal-1 levels (*r* = 0.17, *p* = 0.15; *r* = 0.04, *p* = 0.72, respectively). We then explored whether Gal-1 serum levels correlate with the Erythrocyte Sedimentation Rate (ESR). Notably, we found a very strong positive correlation between Gal-1 serum levels and ESR (Figure 2G, *r* = 0.039, *p* = 0.0006), a blood parameter that indicates the extent of systemic inflammation. Moreover, we also found a positive correlation between Gal-1 serum levels and DAS28 (Disease Activity Score 28) (Figure 2H, *r* = 0.25, *p* = 0.029).

Since RA is a chronic inflammatory disease that aggravates gradually, we also explored whether Gal-1 serum levels could change over time. We found no significant correlation between serum Gal-1 and disease duration (Figure 2I, *r* = 0.18, *p* = 0.15). Additionally, no correlation was found between Gal-1 serum concentrations and patients' age (*r* = 0.03, *p* = 0.80, graph not shown).

In order to broaden our study and given the different roles of Gal-3 in the arthritogenic process, we then examined serum levels of this chimera-type lectin in this patient cohort using a human Gal-3 ELISA kit (R&D Systems; DY1154). Interestingly, RA patients showed significantly lower levels of Gal-3 in circulation compared to control subjects (Figure 2J). Similar to our previous analysis, we categorized RA patients according to their functional status classification and found that serum Gal-3 levels were significantly diminished in functional class I and II compared to controls, but found no statistical differences between controls and class III RA patients (Figure 2K). Moreover, a positive linear trend was found, showing that Gal-3 serum concentrations tended to be higher in classes with higher disease activity (*r* = 0.18, *p* = 0.0037) (Figure 2K). Then, the same correlation analysis applied to Gal-1 and clinical parameters of disease was performed for Gal-3. Although we found no correlation between Gal-3 serum concentrations and VAS (*r* = −0.08, *p* = 0.60), ESR (*r* = −0.15, *p* = 0.31) or DAS28 (*r* = −0.06, *p* = 0.69), a significant positive correlation was detected between Gal-3 levels and HAQ score (Figure 2L, *r* = 0.38, *p* = 0.0098). Furthermore, though circulating Gal-3 levels did not correlate with RA duration (*r* = 0.23, *p* = 0.16), we found a positive correlation with patients' age (Figure 2M, *r* = 0.40, *p* = 0.0062).

Finally, we generated Receiver-Operator Characteristic (ROC) curves in order to assess the ability of Gal-1 and Gal-3 serum levels to discriminate between RA patients and healthy controls. Both Gal-1 and Gal-3 serum levels proved to be good parameters to distinguish patients with established RA from controls, as the area under the ROC curve (AUC) for both parameters was above 0.8 (Gal-1 AUC = 0.82, Gal-3 AUC = 0.88; both *p* < 0.0001) (Figure 2N). Serum Gal-1 concentrations above 60.94 ng/ml (sensitivity = 80.0% and specificity = 73.3%) and serum Gal-3 concentrations below 16.82 ng/ml (sensitivity = 85.42% and specificity = 71.43%) successfully differentiated RA patients from controls.

## Conclusions

Galectins have emerged as amplifiers or silencers of inflammatory responses, capable of orchestrating complex regulatory circuits in innate and adaptive immune cells, as well as in synovial fibroblasts. In this perspective article we summarize relevant data pinpointing the contribution of galectins to the pathogenesis of RA (Figure 1) and report new clinical observations, highlighting the differential regulation of Gal-1 and Gal-3 at the systemic level in RA patients and their association with disease activity (Figure 2).

In two independent cohorts we found increased concentrations of Gal-1 in sera from RA patients compared to control individuals. Elevated levels of this lectin were found in all functional classes of patients and were independent of age and disease duration. To our knowledge, only one study has evaluated circulating Gal-1 levels in RA patients. Xibillé-Friedmann et al described in a cohort of 60 patients that plasma Gal-1 levels were similar in patients and controls; however Gal-1 concentrations were reduced in synovial fluid of patients and correlated with the presence of anti-Gal-1 autoantibodies (40). Although both studies recruited patients under DMARD treatment, differences between them could be related to distinct DMARD used, genetic background and/or environmental factors influencing concentrations of this immunoregulatory lectin. Of note, control subjects from that study exhibited considerably higher levels of Gal-1 in serum (low μg/ml range) compared to our controls and data published by other groups (often ranging in the low ng/ml range) (57–62).

Interestingly, we found a strong correlation between Gal-1 concentrations and ESR, an indicator of systemic inflammation. Similarly, in a previous study, Gal-1 serum levels were significantly increased in classical Hodgkin lymphoma patients who also showed an elevated ERS (57). Accordingly, we observed a positive correlation between serum Gal-1 and DAS-28, a composite score of disease activity derived from examination of 28 joints (number of swollen joints and tender joints) combined with ESR and VAS measurements. Like PD-1, CTLA-4 and other immune checkpoints, Gal-1 expression is upregulated in response to severe inflammatory conditions, acting as an homeostatic mechanism to counterbalance exuberant inflammation (13, 63). Interestingly, nuclear factor (NF)-κB, a transcription factor associated with induction of pro-inflammatory genes, also controls expression of immune inhibitory programs including those involving PD-1 and Gal-1 on T cells (64, 65). Thus, during the peak of inflammation, similar transcriptional mechanisms may operate to activate homeostatic programs that contribute to resolution of inflammatory responses.

The pathogenic role of IL-6 in RA has been widely studied, showing correlation between systemic levels of this cytokine and disease activity (66). Recently, we found that systemic upregulation of IL-6 mobilizes myeloid-derived suppressor cells (MDSCs) which drive Gal-1 production by γδ-T cells (67). In this regard, expansion of MDSCs correlated with disease severity (DAS-28) in RA patients (68, 69). As serum Gal-1 levels positively correlate with inflammation and DAS-28, activation of an “IL-6-MDSCs-Gal-1” axis could also take place in RA. Additional studies should be conducted to verify this hypothesis. On the other hand, a Gal-1-mediated pro-inflammatory signature has been observed in chondrocytes from osteoarthritic patients, suggesting context-dependent regulatory effects of this lectin (70).

Remarkably, Gal-1 and Gal-3 act by cross-linking *N*- and *O*-glycans on the surface of immune cells (15, 71). Since glycosylation is considerably altered in rheumatologic disorders (72), further studies are warranted to examine the relevance of cell surface glycans on immune cells, particularly those implicated in galectin-glycan interactions (complex *N*-glycans, core-2 *O*-glycans and absence of α2,6-sialylated structures) during the evolution of the arthritogenic process in RA patients. In this regard, low levels of galactosylation and sialylation of autoantibodies are associated with disease severity in RA patients (73). Moreover, Pfeifle and colleagues showed that IL-23-activated Th17 cells suppress α2,6-sialylation of IgG through downregulation of the α2,6-sialyltransferase-1 in antibody-producing plasma cells, skewing the balance from anti-inflammatory toward pro-inflammatory responses (74). Further studies aimed at exploring glycosylation patterns of pathogenic cells in RA will contribute to fully elucidate the role of galectins in this pathology.

Finally, we and others observed a positive correlation between Gal-3 levels and HAQ (39). Interestingly, we found lower concentrations of Gal-3 in RA patients compared to controls. In contrast, Issa and colleagues reported augmented Gal-3 serum levels mainly in untreated patients (38, 39). Such discrepancies could be probably due to DMARD and/or corticosteroid treatment in our patient cohorts. Supporting this assumption, glucocorticoid treatment inhibited lipopolysacharides-induced upregulation of Gal-3 in monocytic THP-1 cells (75). Moreover, a significant increase in IgG galactosylation and sialylation was detected in RA patients after initiation of methotrexate therapy, showing reversion to physiologic conditions (76, 77). Thus, low serum Gal-3 levels in combination with augmented Gal-1 expression could influence activation of tolerogenic circuits during RA remission states. Future studies involving treated and untreated RA patients will shed light on how different treatments affect both the glycosylation patterns of inflammatory cells as well as the expression pattern of pro- and anti-inflammatory galectins, leading to activation or de-activation of immune signaling programs.

## Ethics Statement

Patients and controls were informed in detail about the study, and written consent was obtained in accordance with the Declaration of Helsinki. The protocol was approved by Ethics and Research Committees of Hospital de Clínicas José de San Martín, Hospital José Bernardo Iturraspe and Instituto de Biología y Medicina Experimental (IBYME).

## Author Contributions

SPM-H acquired data, analyzed, and interpreted data, supervised the study and wrote the manuscript. PFH interpreted data, and wrote and revised the manuscript. JCS acquired data and revised the manuscript. LGM analyzed and interpreted data, and revised the manuscript. NAP and SMM analyzed data and wrote the manuscript. AMB, JAC, JLM, and GGN managed patients and revised the manuscript. GAR conceived, designed, and supervised the study, interpreted data, and wrote the manuscript.

### Conflict of Interest Statement

The authors declare that the research was conducted in the absence of any commercial or financial relationships that could be construed as a potential conflict of interest.
