# Supplementary material for: Clinical Relevance of Galectin-1 and Galectin-3 in Rheumatoid Arthritis Patients: Differential Regulation and Correlation With Disease Activity
Source: Front Immunol. 2019 Jan 9;9:3057. doi: 10.3389/fimmu.2018.03057 (PMC6333668; doi:10.3389/fimmu.2018.03057)
Supplement: Supplementary file 1 [file Data_Sheet_1.pdf]

Supplementary Material

Supplementary Data

METHODS

Patients

For cohort 1, 32 RA patients from Hospital de Clínicas “Jose de San Martín” (Buenos Aires, Argentine) and 19 sex- and age-matched healthy volunteers were recruited (Table 1, Cohort 1). A second independent cohort of 48 RA patients and 29 sex- and age-matched healthy volunteers was recruited from Hospital “José Bernardo Iturraspe” (Santa Fe, Argentina) to confirm and further extend our initial analysis (Table 1, Cohort 2). All patients were under treatment with at least one disease-modifying anti-rheumatic drug (DMARD) and most of them were also under corticoid treatment, mainly meprednisone.

Clinical assessment

The following disease variables were recorded: Health Assessment Questionnaire (HAQ), Disease Activity Score in 28 joints including erythrocyte sedimentation rate (DAS28) and Visual Analogue Scales (VAS (0–100 mm) of pain.

Determination of Gal-1 and Gal-3

Serum Gal1 was determined using an in-house ELISA as described (56). In brief, high binding 96-well microplates (Costar, Corning) were coated with capture antibody (2 µg/ml purified rabbit anti–Gal-1 polyclonal IgG) in 0.1 M sodium carbonate, pH 9.5. After incubation for 18 h at 4°C, wells were rinsed three times with washing buffer (0.05% Tween-20 in PBS) and incubated for 1 h at room temperature with blocking solution (2% BSA in PBS). One hundred µl of samples and standards were diluted in 1% BSA and incubated for 18 h at 4°C. Plates were then washed and incubated with 100 ng/ml biotinylated detection antibody (purified rabbit anti–Gal-1 polyclonal IgG) for 1 h. Plates were rinsed three times before adding 0.3 µg/ml HRP-labeled streptavidin (Sigma-Aldrich) for 30 min. After washing, 100 µl TMB solution (0.1 mg/ml tetramethylbenzidine and 0.06% H_2O_2 in citrate-phosphate buffer, pH 5.0) was added to the plates. The reaction was stopped by adding 4N H2SO4. Optical densities were determined at 450 nm in a Multiskan MS microplate reader (Thermo Fisher Scientific). A standard curve ranging from 2.5 to 160 ng/ml recombinant Gal-1 was run in parallel.

Serum Gal-3 levels were determined with the human Gal-3 DuoSet ELISA kit (DY1154, R&D Systems), following manufacturer´s instructions.

Statistical Analysis

All variables analyzed were tested for Gaussian distribution with D'Agostino and Pearson omnibus normality test. For comparisons between two groups, unpaired t test with Welch´s correction or Mann-Whitney test were applied as appropriate. For comparisons between more than two groups, Kruskal-Wallis test was applied. For correlation analysis, Pearson or Spearman correlation tests were applied as appropriate. To determine the capability of Gal-1 and Gal-3 serum level measurements to discriminate between RA patients and controls, ROC curves were generated.
